# Supplementary material for: Aedes aegypti Malpighian tubules are immunologically activated following systemic Toll activation
Source: Parasit Vectors. 2022 Dec 15;15:469. doi: 10.1186/s13071-022-05567-2 (PMC9753289; doi:10.1186/s13071-022-05567-2)
Supplement: Supplementary file 9 — Additional file 9: Figure S1. Gene set enrichment analysis (GSEA) of Malpighian tubule transcriptional responses to Toll activation. GSEA was performed with four manually curated gene sets from previously published transcriptomic studies which showed significant associations in our Malpighian tubule transcriptomic comparisons. Each panel represents the association of one gene set where the y-axis shows the enrichment score (ES), and the x-axis shows the log2FC rank of all genes detected in the transcriptome. Black ticks represent where genes in the respective gene list fall on the continuum of ranked genes. ES and the corrected P-value (false discovery rate [FDR]) for each gene set shown. ES scores indicate genes are enriched in those that are upregulated (positive ES) or downregulated (negative ES) following dsCactus treatment. Full statistical description of all lists can be found in Additional file: Table S5. [file 13071_2022_5567_MOESM9_ESM.pdf]

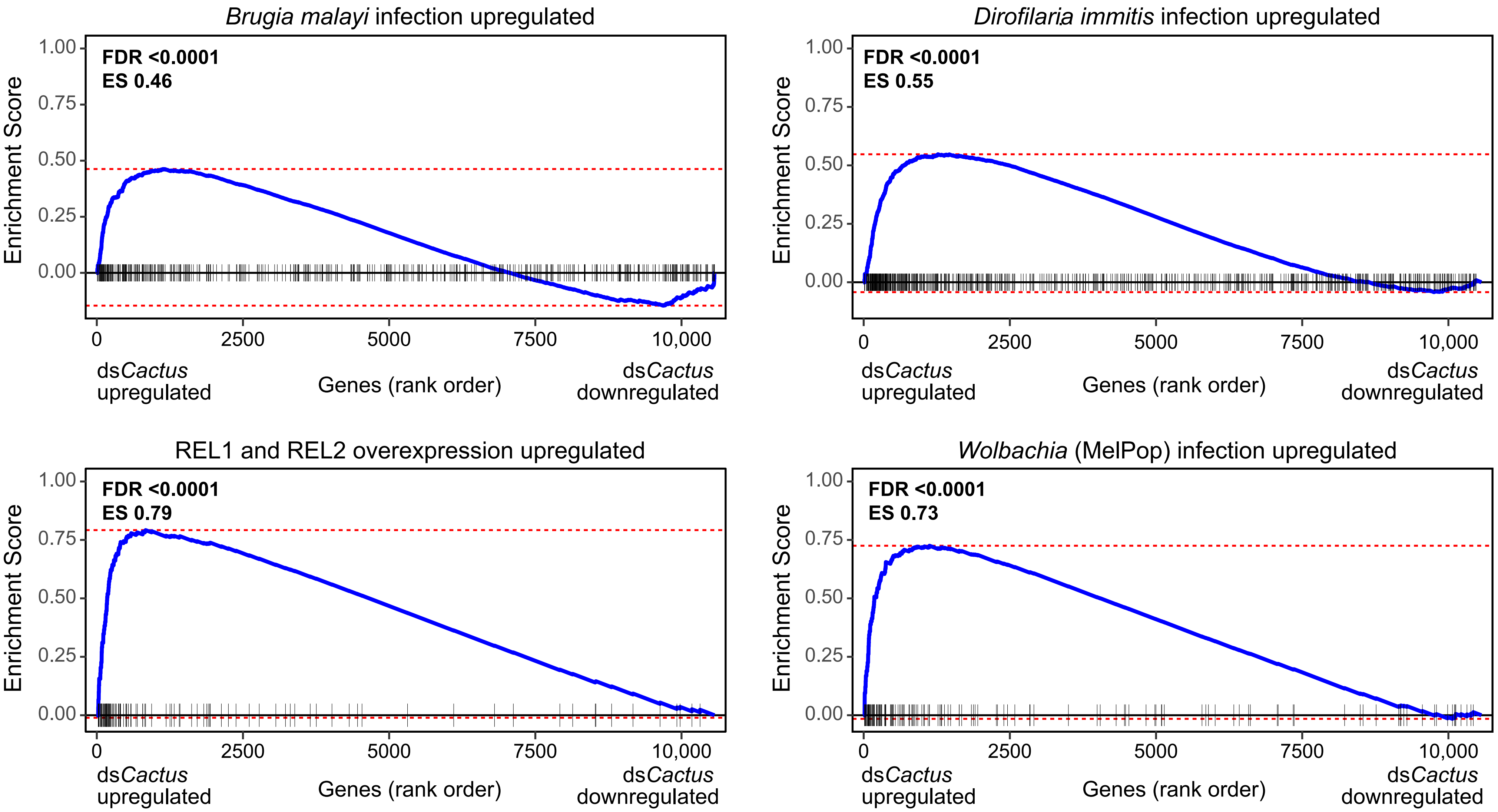
Brugia malayi infection upregulated
FDR <0.0001
ES 0.46
Enrichment Score
1.00
0.75
0.50
0.25
0.00
0
2500
5000
7500
10,000
dsCactus upregulated
Genes (rank order)
dsCactus downregulated
Dirofilaria immitis infection upregulated
FDR <0.0001
ES 0.55
REL1 and REL2 overexpression upregulated
FDR <0.0001
ES 0.79
Wolbachia (MelPop) infection upregulated
FDR <0.0001
ES 0.73

Figure S1
