# Supplementary figures and images for: Aedes aegypti Malpighian tubules are immunologically activated following systemic Toll activation
Source: Parasit Vectors. 2022 Dec 15;15:469. doi: 10.1186/s13071-022-05567-2 (PMC9753289; doi:10.1186/s13071-022-05567-2)

## Sample correlation

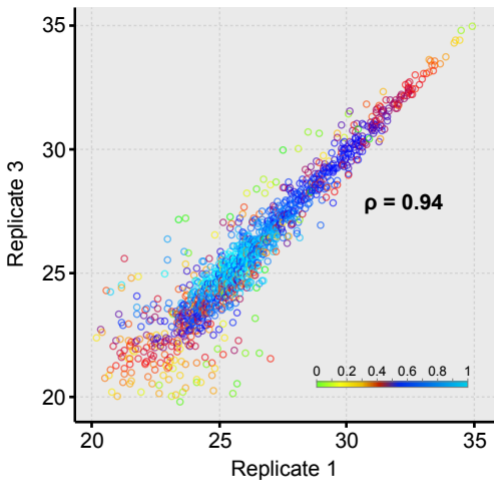

Replicate 1 vs 2 ( $\rho = 0.91$ )

Replicate 2 vs 3 ( $\rho = 0.93$ )

**Figure S2**

Supplement: Supplementary file 10 — Additional file 10: Figure S2. Hemolymph proteomic replicates are highly correlated. Correlation analysis to demonstrate consistency between hemolymph mass spectrometry proteomic replicates. Replicate 1 (x-axis) is shown against replicate 3 (y-axis) with the correlation coefficient ρ value (0.94) shown on the graph. Below the graph are the ρ values for the other replicate comparisons, which are also highly correlated to each other. [file 13071_2022_5567_MOESM10_ESM.pdf]

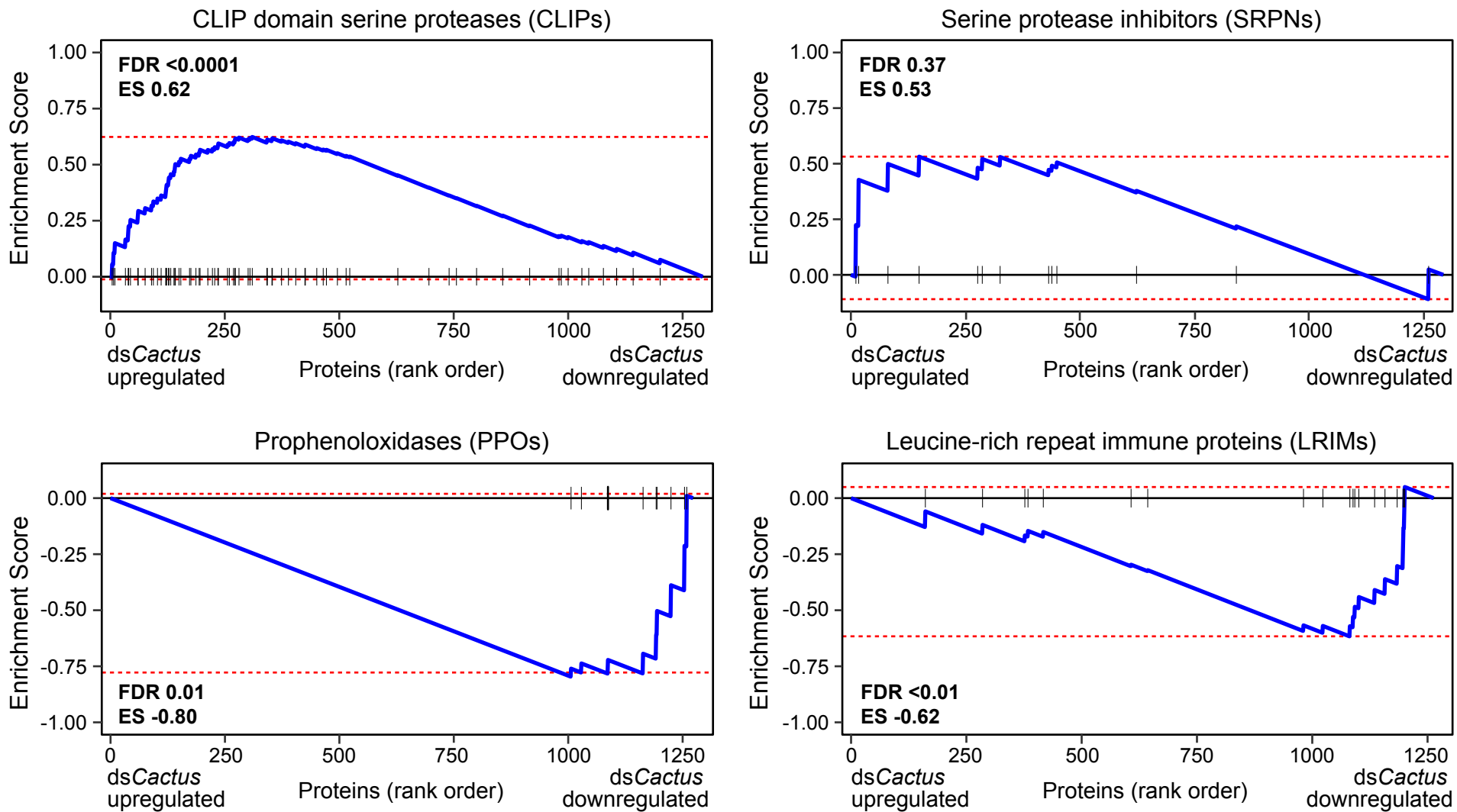

Figure S3

Supplement: Supplementary file 11 — Additional file 11: Figure S3. Protein set enrichment analysis of hemolymph response to Toll activation. The y-axis shows the enrichment score (ES) and the x-axis shows the log2FC rank of all proteins detected in the hemolymph proteome. Black ticks represent where genes of the respective proteins from curated lists fall on the continuum of ranked proteins. Full statistical description of all lists can be found in Additional file: Table S5. [file 13071_2022_5567_MOESM11_ESM.pdf]
